# Maternal Exercise during Pregnancy Increases BDNF Levels and Cell Numbers in the Hippocampal Formation but Not in the Cerebral Cortex of Adult Rat Offspring

**DOI:** 10.1371/journal.pone.0147200

**Published:** 2016-01-15

**Authors:** Sérgio Gomes da Silva, Alexandre Aparecido de Almeida, Jansen Fernandes, Glauber Menezes Lopim, Francisco Romero Cabral, Débora Amado Scerni, Ana Virgínia de Oliveira-Pinto, Roberto Lent, Ricardo Mario Arida

**Affiliations:** 1 Departamento de Fisiologia, Universidade Federal de São Paulo (UNIFESP), São Paulo—SP, Brazil; 2 Hospital Israelita Albert Einstein, São Paulo—SP, Brazil; 3 Universidade de Mogi das Cruzes (UMC), Mogi das Cruzes—SP, Brazil; 4 Disciplina de Neurologia Experimental, Universidade Federal de São Paulo (UNIFESP), São Paulo—SP, Brazil; 5 Instituto de Ciências Biomédicas, Universidade Federal do Rio de Janeiro (UFRJ), Rio de Janeiro—RJ, Brazil; Université Pierre et Marie Curie, FRANCE

## Abstract

Clinical evidence has shown that physical exercise during pregnancy may alter brain development and improve cognitive function of offspring. However, the mechanisms through which maternal exercise might promote such effects are not well understood. The present study examined levels of brain-derived neurotrophic factor (BDNF) and absolute cell numbers in the hippocampal formation and cerebral cortex of rat pups born from mothers exercised during pregnancy. Additionally, we evaluated the cognitive abilities of adult offspring in different behavioral paradigms (exploratory activity and habituation in open field tests, spatial memory in a water maze test, and aversive memory in a step-down inhibitory avoidance task). Results showed that maternal exercise during pregnancy increased BDNF levels and absolute numbers of neuronal and non-neuronal cells in the hippocampal formation of offspring. No differences in BDNF levels or cell numbers were detected in the cerebral cortex. It was also observed that offspring from exercised mothers exhibited better cognitive performance in nonassociative (habituation) and associative (spatial learning) mnemonic tasks than did offspring from sedentary mothers. Our findings indicate that maternal exercise during pregnancy enhances offspring cognitive function (habituation behavior and spatial learning) and increases BDNF levels and cell numbers in the hippocampal formation of offspring.

## Introduction

Brain development is a highly plastic process that in humans starts in utero and extends at least through late adolescence. Events that happen during this period can modulate the functional maturation of the brain and determine its lifelong integrity [1]. In this context, it has been observed that environmental stimuli such as maternal physical exercise may favor brain development [2,3,4,5,6,7]. Clinical evidences indicate that the practice of exercise during pregnancy positively influences fetal health and improves cognitive performance in early childhood [2,8,9]. Children of women who exercised regularly throughout their pregnancies had better performance on tests of general intelligence and oral language skills than did children of sedentary mothers [2].

In laboratory animals, studies have also shown that exercise during pregnancy affects various brain functions in pups [3,4,5,6,7] and mitigates the effects of an Alzheimer-like pathology in adult offspring [10]. These findings result from experiments in which several cognitive tests and models of physical exercise were applied, including forced running on a treadmill, forced swimming, and voluntary wheel running. For example, in a study conducted by Parnpiansil and collaborators [3], rats submitted to treadmill exercise during pregnancy had pups with better spatial learning scores in a multiple T maze test, compared to pups born of sedentary rats. It was also noted that maternal running on a treadmill during pregnancy improved memory performance of offspring in a step-down avoidance task, in comparison to the offspring of sedentary rats [5]. Other work showed that pups born from rats that swam during their pregnancies exhibited higher memory performance in a step-down avoidance task [4]. Additionally, it was shown that both forced and voluntary maternal exercise during pregnancy increased pups’ learning in a water maze test [6]. In a more recent study [7], the offspring of rats with free access to a running wheel throughout gestation were better at discriminating between novel and familiar objects in a memory task than the offspring of sedentary rats.

Taken together, these data suggest that maternal exercise during gestation enhances offspring brain function throughout life. Nevertheless, the mechanisms through which maternal exercise might promote such effects are not well understood. A possible explanation is that exercise during pregnancy can affect mechanisms that control neuronal function during development and afterwards, resulting in improved cognitive functioning of offspring. In the present study, therefore, we determined levels of brain-derived neurotrophic factor (BDNF) and absolute cell numbers in the hippocampal formation and cerebral cortex of rat pups born from mothers exercised during pregnancy. In addition, we evaluated the cognitive abilities of the pups in different behavioral paradigms (exploratory activity and habituation in open field tests, spatial memory in a water maze test, and aversive memory in a step-down inhibitory avoidance task).

## Materials and Methods

### Exercise Paradigm

Eight-week-old pregnant Wistar rats were used in this study. Rats were housed individually in plastic home cages. The colony room was maintained at 21 ± 2°C with a 12-h light/dark schedule (lights on at 7 a.m.) and *ad libitum* food and water throughout the experiments. Gestational day was timed from the appearance of a vaginal plug after mating. Pregnant rats at gestational day 1 (G1) were randomly assigned into two groups: exercise (n = 17) and control (n = 15). Rats from the exercise group were submitted to physical exercise on a treadmill (AVS Projetos). We chose treadmill running because the intensity and duration of exercise can be easily controlled, unlike voluntary wheel running [11]. Physical training was performed once a day from G1 to G20, between 9:00 and 10:00h a.m. Exercise sessions started with a 3-min warm-up at 8 m/min, and electric shocks (1 mA) were used sparingly to motivate the rats to run. Running time and speed were gradually increased from 10 min at 10 m/min during the first sessions to 30 min at 12 m/min over the following training days. Pregnant rats from the control group were transferred to the experimental room and kept on a stopped treadmill during the same time and circadian periods employed for the exercise group. At G21, pregnant rats from all groups were maintained at rest (non-training) in our laboratory to monitor the birth of the litter. After birth, some female pups were removed from the litter to maintain an equal number of eight pups per mother. This procedure was performed to avoid possible nutritional changes in the litter as well as to achieve our objective of analyzing only male offspring. Removed female pups were allocated to other mothers, who were not related to the present study. Male pups from exercise and control groups were housed with their mother in individual cages until weaning at postnatal day 21 (P21). After weaning, the offspring were housed in cages with same-sex littermates in groups of five rats. The colony room was maintained under the same conditions as described above. All experimental protocols were approved by the ethics committee of the Universidade Federal de São Paulo (#0607/09).

### BDNF Analysis

Cortical and hippocampal BDNF levels of offspring from the exercise (n = 8) and control (n = 9) groups were evaluated at P60. Analysis of BDNF protein was performed only in male offspring. The cerebral cortex and hippocampal formation of the pup were dissected out of the removed brain immediately after decapitation and homogenized in 0.01 M Tris hydrochloride (pH 7.6) containing 5.8% sodium chloride, 10% glycerol, 1% Nonidet P40 (NP-40), 0.4% ethylenediamine tetraacetic acid (EDTA), and protease inhibitors. Samples were sonicated and stored at -80°C. BDNF analysis was performed using the ELISA kit E-max^®^ (Promega) according to the manufacturer's recommendations. Cortical and hippocampal samples from exercise and control groups previously stored at -80°C were transferred to a 96-well plate (Corning Costar) coated with anti-BDNF (incubated overnight at 4°C without shaking) and kept at room temperature for 2 h. After this period, the plate was washed with Tris-buffered saline Tween-20 (TBS-T) and incubated with the following antibodies: anti-human BDNF (1:500) for 2 h and conjugate anti-IgY HRP (1:200) for 1 h. After these procedures, the color reaction with tetramethyl benzidine was quantified in a plate reader at 450 nm (Quick Elisa). Values were reported in pg/ml.

### Quantification of Cortical and Hippocampal Cells

The total number of neuronal and non-neuronal cells in the cerebral cortex and hippocampal formation from studied groups was investigated at P60. Cortical and hippocampal quantification was performed only in male offspring (n = 7 in each group), and total numbers of cells were estimated as described previously using the isotropic fractionator method [12]. Briefly, the animal was deeply anesthetized (Thionembutal, 50 mg/kg, i.p.) and perfused transcardially with a solution of 0.01 M phosphate-buffered saline (PBS), followed by a solution containing 4% formaldehyde in 0.1 M phosphate buffer (PB), pH 7.4. After perfusion, the brain was immediately removed from the skull and postfixed with 4% paraformaldehyde in PB for 24 h. The cortex and the hippocampal formation were dissected and chemomechanically dissociated in a saline solution with 0.1% Triton X-100 to achieve an isotropic suspension of isolated nuclei kept homogeneous by agitation. The cerebral cortex and hippocampal formation (Ammon's horn and dentate gyrus) were dissected by mean of consistent anatomical landmarks (the same criteria for dissection used by Herculano-Houzel and Lent [12]). The cerebral cortex comprised all regions dorsolateral to the olfactory tract, excluding the hippocampal formation, and was dissected from each hemisphere by peeling it away from the striatum and other subcortical structures. Since none significant difference in cortical (p = 0.3445) and hippocampal (p = 0.1415) mass was detected between exercise (cortex = 0.591 ± 0.033 g; hippocampus = 0.083 ± 0.005 g) and control (cortex = 0.505 ± 0.062 g; hippocampus = 0.075 ± 0.001 g) groups, no correction was used. Moreover, there was no significant difference between the exercise (cortex = 10.54 ± 0.20 ml; hippocampus = 9.15 ± 0.36 ml) and control (cortex = 10.67 ± 0.37 ml; hippocampus = 9.10 ± 0.26 ml) groups in final volume of dissociation solution (saline solution with 0.1% Triton X-100) in cerebral cortex (p = 0.775) and hippocampal formation (p = 0.775) of offspring. The total number of cells was estimated by determining the number of nuclei in small aliquots stained with the fluorescent DNA marker 4’-6-diamidino-2-phenylindole dihydrochloride (DAPI) under a Zeiss Axiovert 100 microscope with a 40x objective, using a hemocytometer (Neubauer chamber) for quantification. To determine neuronal and non-neuronal cell numbers, the samples were then incubated with a primary antibody against the neuron-specific nuclear protein (NeuN; 1:200; Chemicon) at 4°C overnight and, subsequently, with a secondary antibody (1:300) conjugated to AlexaFluor^®^ 555 diluted in PBS with DAPI and 10% normal goat serum for 2 h. The neuronal fraction in each sample was estimated by counting NeuN-labeled nuclei in at least 500 DAPI-stained nuclei and the number of non-neuronal nuclei was obtained by subtraction. The counting was performed independently by two investigators.

### Behavioral Analyses

Exploratory activity, habituation, and spatial and aversive memories of the offspring (only male) from the exercise (n = 65) and control (n = 66) groups were evaluated from P60 to P69. Exploratory activity and habituation were tested in an open field apparatus (n = 35 for exercise group and n = 36 for control group). Spatial and aversive memories were tested in a water maze and by an inhibitory avoidance task, respectively (n = 30 in each group). Some of the offspring (n = 15 in each group) were evaluated in the water maze from P60 to P67 and by the inhibitory avoidance task from P68 to P69. The rest of the offspring (n = 15 in each group) were evaluated by inhibitory avoidance from P60 to P61 and in the water maze from P62 to P69. This procedure was performed to ensure that the inhibitory avoidance results were not influenced by the water maze task and vice versa. All behavioral procedures were conducted between 2:00 and 5:00 p.m. in a sound-isolated room.

### Open Field Test

An open field apparatus was used to evaluate both exploratory activity and habituation. The offspring was exposed to a cylindrical arena (100 x 35 cm) of white acrylic polyvinyl. The floor of the arena was divided into 13 quadrants of equal area by lines. The animal was gently placed on the central quadrant and then left alone to explore the arena for 5 min (n = 14 for the exercise group and n = 14 for the control group), and a different set of animals was investigated for 10 min (n = 21 for the exercise group and n = 22 for the control group). Crossings of quadrant lines were counted and used as measures of locomotion and exploration. Immediately after this procedure, the animals were taken back to their home cage and 24 h later they were reexposed to the open field apparatus for 5 min and 10 min, as before. Every crossing of quadrant lines was again counted. The decrease in the number of crossings between the two sessions was considered as a measure of habituation, a type of nonassociative memory [13]. It is important to point out that habituation test was performed in two subgroups of animals that were investigated independently for 5 or 10 min.

### Water Maze Test

The water maze task was similar to that described by Morris [14]. The water maze consisted of a black circular pool (200 x 50 cm) conceptually divided into four equal imaginary quadrants (quadrants 1–4). The water temperature was maintained between 21–25°C. A black circular platform (12 x 25 cm) was placed 1.5 cm under the water surface in the center of quadrant 3. Various objects (frames, pictures) were fixed on the walls of the experimental room to be used as reference points. Two groups of offspring animals (n = 15 in each group) were placed in the water maze and allowed to search for the platform for 60 sec and 120 sec, respectively. Over seven days (P60–P66), they performed one trial per day and began the test at different points (labeled North, South, East and West) on each day. A video-camera (Sony) fixed above the water maze recorded the experiments. The time to reach the platform (latency) of each animal in the maze was analyzed using EthoVision software (Noldus).

A day after the end of the last session in the water maze, the platform was removed for a probe trial. The time spent in each of the four imaginary quadrants was recorded. The amount of time spent in quadrant 3 was used as a measure of offspring memory of the platform location [15,16].

### Inhibitory Avoidance Test

The inhibitory avoidance apparatus consisted of two acrylic boxes, each measuring 21 x 26 x 27.5 cm, connected by a sliding door. A box with white acrylic walls was designated as the safe compartment, whereas the other, with black acrylic walls, was the aversive compartment. The floor of the apparatus was made of parallel metallic rods (0.4 cm in diameter) separated by a distance of 1.2 cm from each other and connected to an electric shock generator. During the task training, each animal was placed in the safe compartment with the sliding door closed. Ten seconds later, the door was opened. As soon as the animal crossed to the aversive compartment with its four paws, the door was closed, the latency to enter was recorded, and the animal received one footshock. Considering that high-intensity shock can suppress scopolamine-induced amnesia in rats [17], we tested the aversive memory of our animals with two footshock intensities: one group of offspring animals was tested with 0.4 mA for 1 sec (n = 14 for the exercise group and n = 13 for the control group), and a different set of animals was tested with 0.6 mA for 1 sec (n = 15 for both the exercise and control groups). After the shock, the animal was removed from the apparatus and returned to the home cage. In the test phase, 24 h later, each animal was first placed in the safe compartment of the apparatus, and the sliding door was opened 10 sec later. The latency to cross to the aversive compartment was recorded. Each animal was allowed 540 sec to cross to the aversive compartment. If it failed to do so, the animal was removed from the apparatus, and a latency of 540 sec was recorded.

### Statistical Analyses

Statistical analyses were conducted using Student’s *t* test, the chi-square test, two-way ANOVA or one-way ANOVA with repeated measures (software version 17.0, SPSS Inc., Chicago, IL). Differences were considered significant when p < 0.05. Results are presented as mean and standard error of the mean (± SEM). All original data are within S1 Table.

## Results

### Maternal Weight Gain

All pregnant rats were weighed every three days between 07:00 and 10:00 a.m. from G1 to G21. In addition, pregnant rats in the exercise group were always weighed before physical training sessions. Animals in both exercise and control groups gained weight during the pregnancy (F_(6,210)_ = 300.6; p < 0.0001), with no significant differences between groups (F_(1,210)_ = 2.55; p = 0.115).

### Number and Gender of Pups

After birth, pups were counted and separated according to sex. Each litter of the exercise group had an average of 11 pups, whereas control group litters averaged 12 pups. Regarding gender, the average was 6 males and 5 females for the exercise group and 6 males and 6 females for the control group. These differences in number (t_(22)_ = 0.83; p = 0.41) and gender (χ^2^_(1)_ = 0.38; p = 0.53) of pups were not statistically significant.

### BDNF Levels

**Fig 1:**
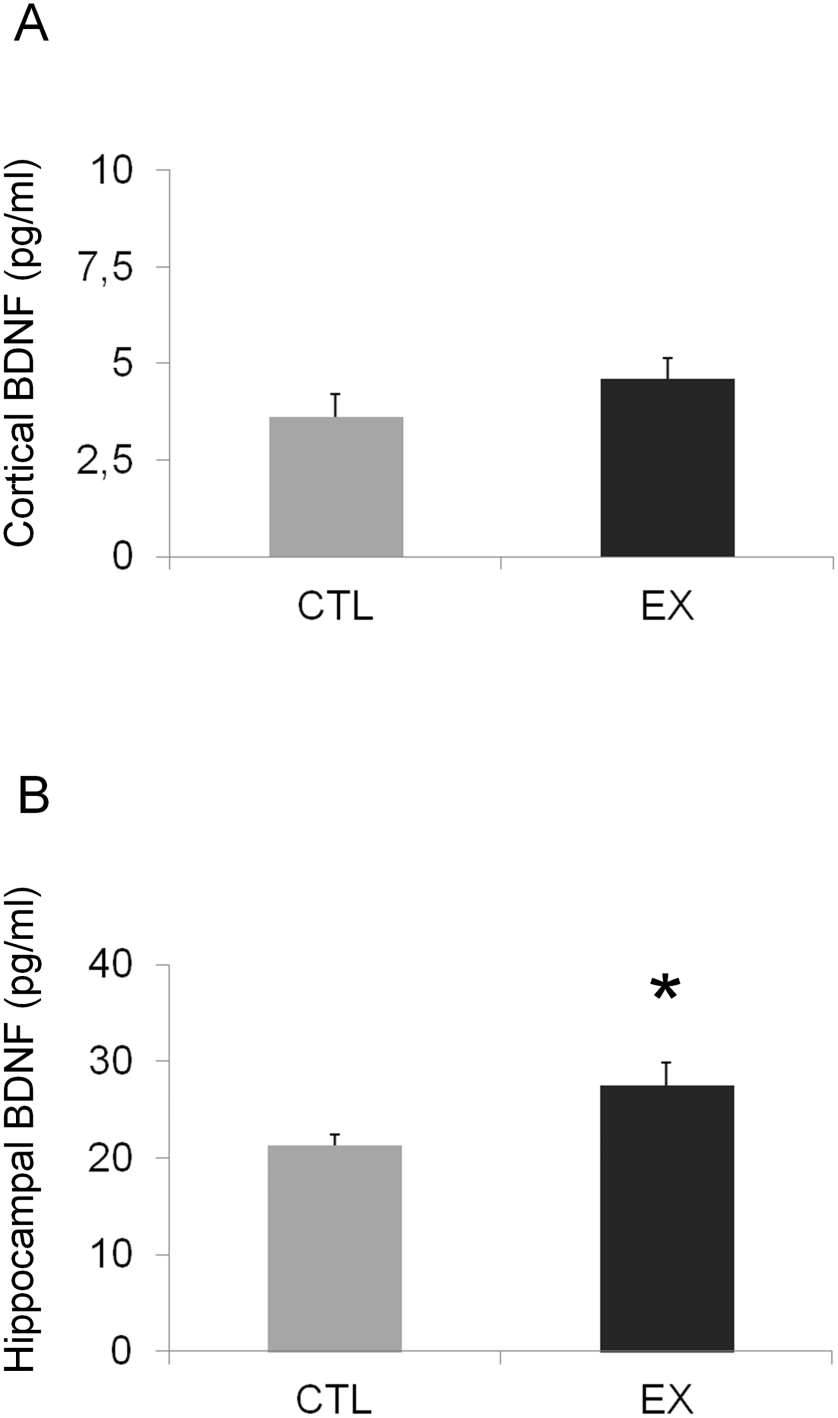
BDNF is increased in the hippocampal formation of offspring of rats exercised during pregnancy. BDNF levels in the cortex and hippocampal formation of offspring from the exercise group (EX; n = 8) and control group (CTL; n = 9) at P60. No significant difference in cortical BDNF levels was observed between the studied groups (A). On the other hand, a significant increase in hippocampal BDNF levels was detected in the EX group when compared to the CTL group (*p < 0.05 by Student's *t* test) (B).

Cortical and hippocampal BDNF levels were investigated at P60. In the cortex, no significant difference in BDNF levels was observed between the exercise and control groups (t_(15)_ = 1.24; p = 0.23) (Fig 1A). However, a significant increase in BDNF level was detected in the hippocampal formation of offspring animals belonging to the exercise group, in comparison to control offspring (t_(15)_ = 2.40; p = 0.029) (Fig 1B).

### Number of Cortical and Hippocampal Cells

**Fig 2:**
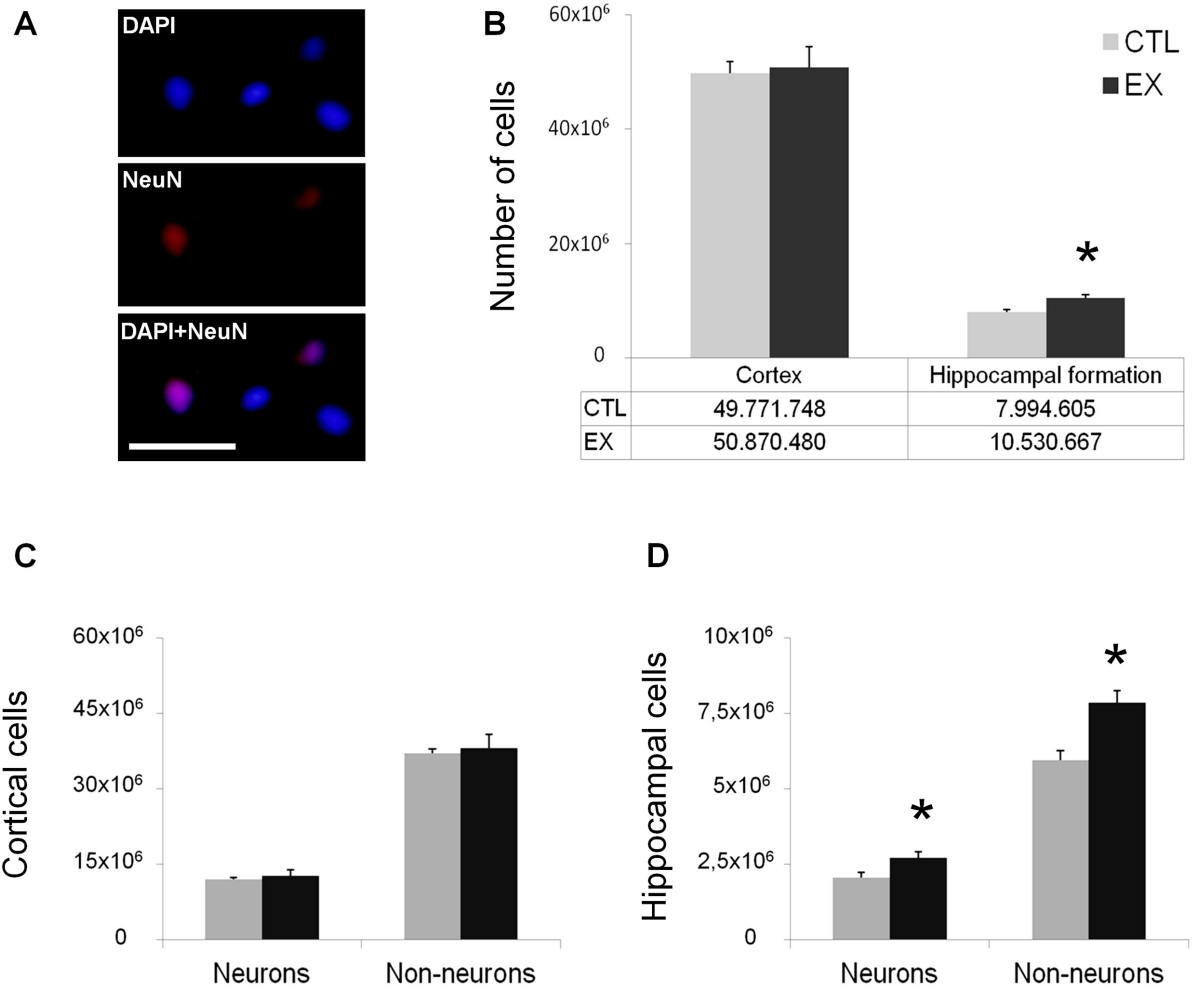
Neuronal and non-neuronal cell numbers were increased in offspring of rats exercised during pregnancy. Number of cortical and hippocampal cells in offspring of the exercise group (EX; n = 7) as compared with the control group (CTL; n = 7), both at P60. (A) Nuclei stained with DAPI (blue) and NeuN (red) as visualized by fluorescence microscopy. Calibration bar = 0.05 mm. (B) Increase in total number of cells in the hippocampal formation but not in the cerebral cortex of offspring in the EX group relative to the CTL group. (C) Lack of significant difference between the EX and CTL groups in number of both neurons and non-neurons in the cerebral cortex. (D) Increase in number of hippocampal neurons and non-neurons in the EX group relative to the CTL group (*p < 0.05 by Student's *t* test).

Numbers of cortical and hippocampal cells were assessed at P60 by the isotropic fractionator method, an unbiased technique designed to determine the absolute cellular composition of different brain regions [12]. To quantify the number of cells, all nuclei were labeled with DAPI, while neuronal nuclei were specifically labeled with NeuN. Counted aliquots showed isolated, round nuclei without clusters (Fig 2A). No significant difference between exercise and control groups was detected in total number of cortical cells (t_(12)_ = 0.47; p = 0.64) (Fig 2B). When neuronal and non-neuronal cell counts were evaluated separately, no significant difference in the number of either cortical cell type was observed in exercise group animals, as compared to control group animals (t_(12)_ = 0.62; p = 0.54 for neuronal nuclei; t_(12)_ = 0.40; p = 0.69 for non-neuronal nuclei) (Fig 2C). In the hippocampal formation, however, exercise group offspring showed a significant increase in total number of cells (t_(12)_ = 3.31; p = 0.006) (Fig 2B) as well as in the numbers of neuronal (t_(12)_ = 2.47; p = 0.02) and non-neuronal cells (t_(12)_ = 3.65; p = 0.003) (Fig 2D).

### Exploratory Activity

**Fig 3:**
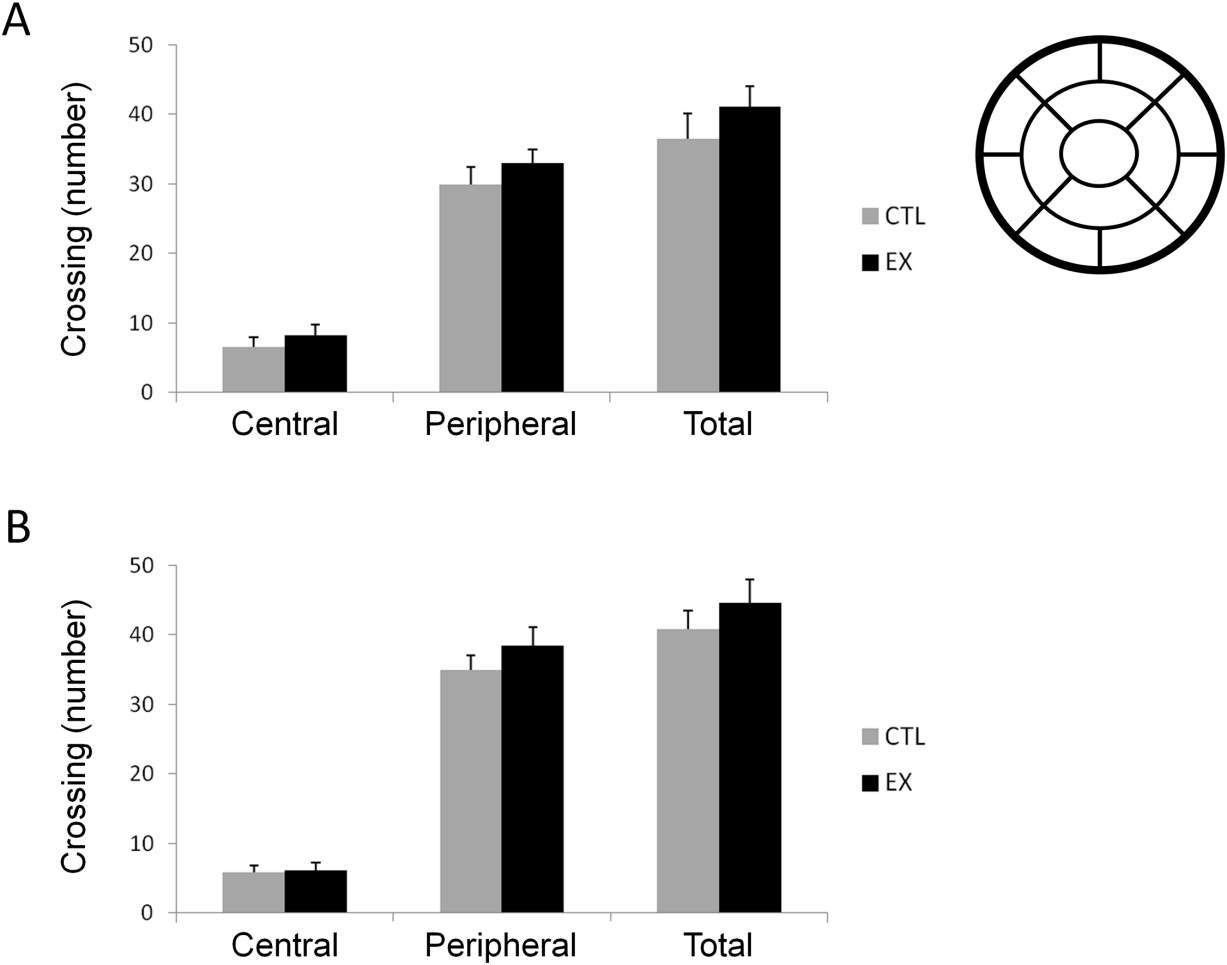
Exercise during pregnancy does not influence exploratory activity of offspring at P60. Open field quadrants (top right) and exploratory activity in offspring from the exercise group (EX; n = 14 for 5 min investigation and n = 21 for 10 min investigation) and control group (CTL; n = 14 for 5 min investigation and n = 22 for 10 min investigation) at P60. Offspring animals were monitored for 5 (A) and 10 (B) minutes. Exploratory activity of offspring is expressed as number of quadrants crossed (locomotion units) for both the 5 central quadrants (central) and the 8 peripheral quadrants (peripheral). Total locomotion corresponds to the sum of central and peripheral locomotion. Student's *t* test revealed no significant difference in exploratory activity between the studied groups for either 5 or 10 min of investigation.

Exploratory activity was measured in an open field apparatus at P60. No significant difference between exercise and control groups was detected for 5 min (t_(26)_ = 0.77; p = 0.44 for central locomotion; t_(26)_ = 0.95; p = 0.35 for peripheral locomotion; t_(26)_ = 1.00; p = 0.32 for total locomotion) or 10 min of investigation (t_(41)_ = 0.17; p = 0.85 for central locomotion; t_(41)_ = 1.01; p = 0.31 for peripheral locomotion; t_(41)_ = 0.85; p = 0.39 for total locomotion) (Fig 3A and 3B).

### Habituation

**Fig 4:**
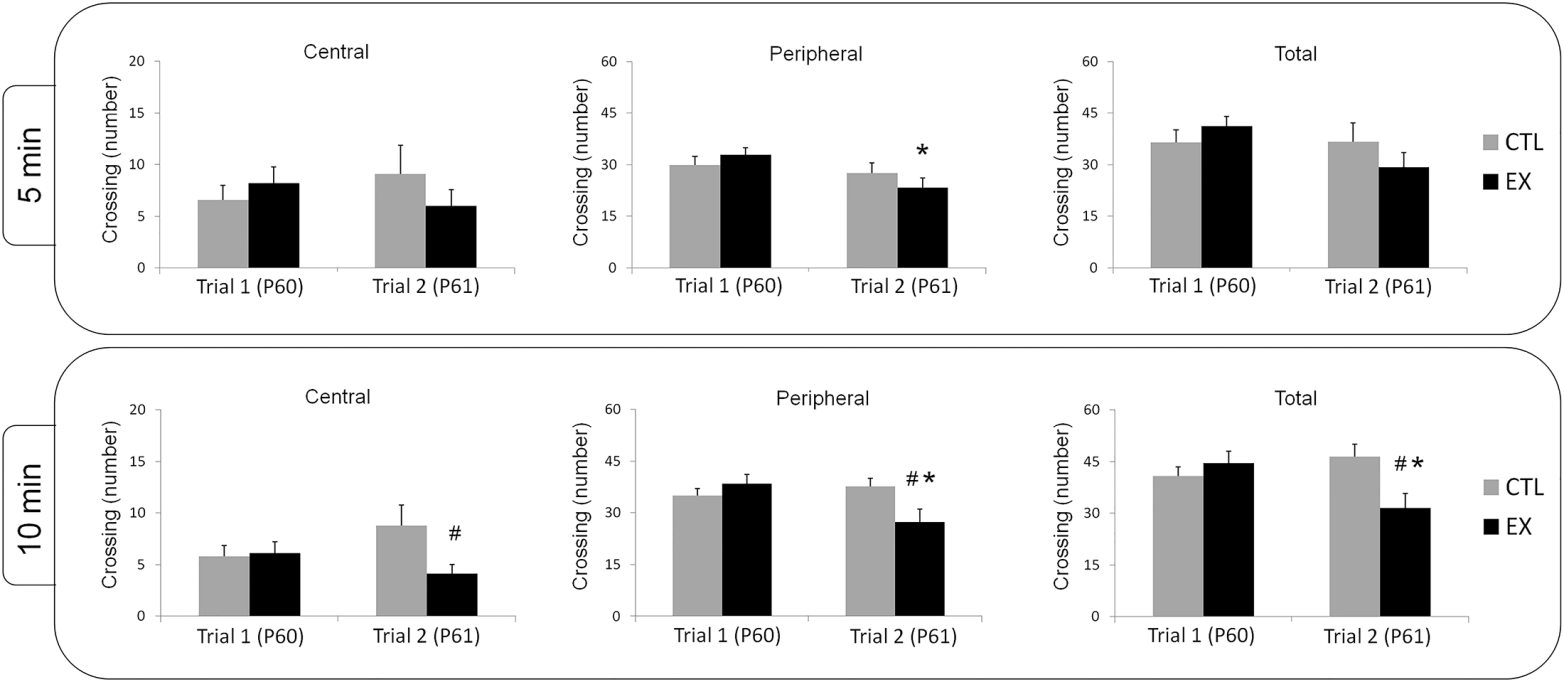
Decreased exploratory activity in the second day trial in offspring of exercised mothers indicates habituation. Exploratory activity in offspring from the exercise group (EX) and control group (CTL) investigated for 5 min (n = 14 in each group) and 10 min (EX n = 22 and CTL; n = 21) at P60 and P61. For both the 5- and 10-min investigation periods, a significant decrease in peripheral locomotion was detected in the EX group between the first and second trial (*p < 0.05; one-way ANOVA with repeated measures; Bonferroni post hoc). No significant difference in central, peripheral, or total locomotion was found in the CTL group between the first and the second trial. In the second trial of the 10-min investigation period, central, peripheral, and total locomotion were lower in the EX group relative to the CTL group (^#^p < 0.05; one-way ANOVA with repeated measures; Bonferroni post hoc).

Habituation was measured in the open field apparatus 24 h after the exploratory test (P61). Two different groups of offspring were submitted to 5 or 10 minute of test. In offspring investigated over 5 min, two-way ANOVA demonstrated no significant effects of day (F_(1,26)_ = 0.008; p = 0.931), group (F_(1,25)_ = 0.109; p = 0.743), or their interaction (F_(1,26)_ = 2.062; p = 0.163) were found for central locomotion. For peripheral locomotion, we detected a significant effect of day (F_(1,26)_ = 8.266; p = 0.008), whereas neither group (F_(1,26)_ = 0.047; p = 0.830) nor their interaction (F_(1,26)_ = 3.047; p = 0.093) had a significant effect. When Bonferroni post hoc analysis was performed, a significant reduction in peripheral locomotion was noted between the first and second trials for the exercise group (p = 0.003). No significant difference in peripheral locomotion was found between the first and second trials for the control group. Analyzing total locomotion, we did not observe a significant effect of day (F_(1,26)_ = 2.958; p = 0.097), group (F_(1,26)_ = 0.078; p = 0.783), or their interaction (F_(1,26)_ = 3.180; p = 0.086) (Fig 4).

In offspring investigated over 10 min, two-way ANOVA demonstrated no significant effects of day (F_(1,41)_ = 0.190; p = 0.666) or group (F_(1,41)_ = 2.067; p = 0.158) were noted for central locomotion, although a significant interaction was found (F_(1,41)_ = 4.545; p = 0.039). For peripheral locomotion, we found significant effects of day (F_(1,41)_ = 4.835; p = 0.034) and the day×group interaction (F_(1,41)_ = 12.742; p = 0.001), whereas the group effect was not statistically significant (F_(1,41)_ = 1.005; p = 0.322). For total locomotion, no significant effect of day (F_(1,41)_ = 2.318; p = 0.136) or group (F_(1,41)_ = 1.632; p = 0.209) was found, although a significant interaction was observed (F_(1,41)_ = 14.483; p < 0.001). Bonferroni post hoc analysis indicated a significant decrease in peripheral (p < 0.001) and total locomotion (p = 0.001) between the first and second trials in the exercise group. No significant differences in central, peripheral, or total locomotion were found between the first and second trials for the control group. In the second trial, the exercise group had significantly reduced central (p = 0.042), peripheral (p = 0.022), and total locomotion (p = 0.010) in comparison to the control group (Fig 4).

Taken together, these results show that offspring from exercised mothers had better cognitive performance in nonassociative mnemonic tasks in comparison with the offspring from sedentary mothers.

### Spatial Learning and Memory

**Fig 5:**
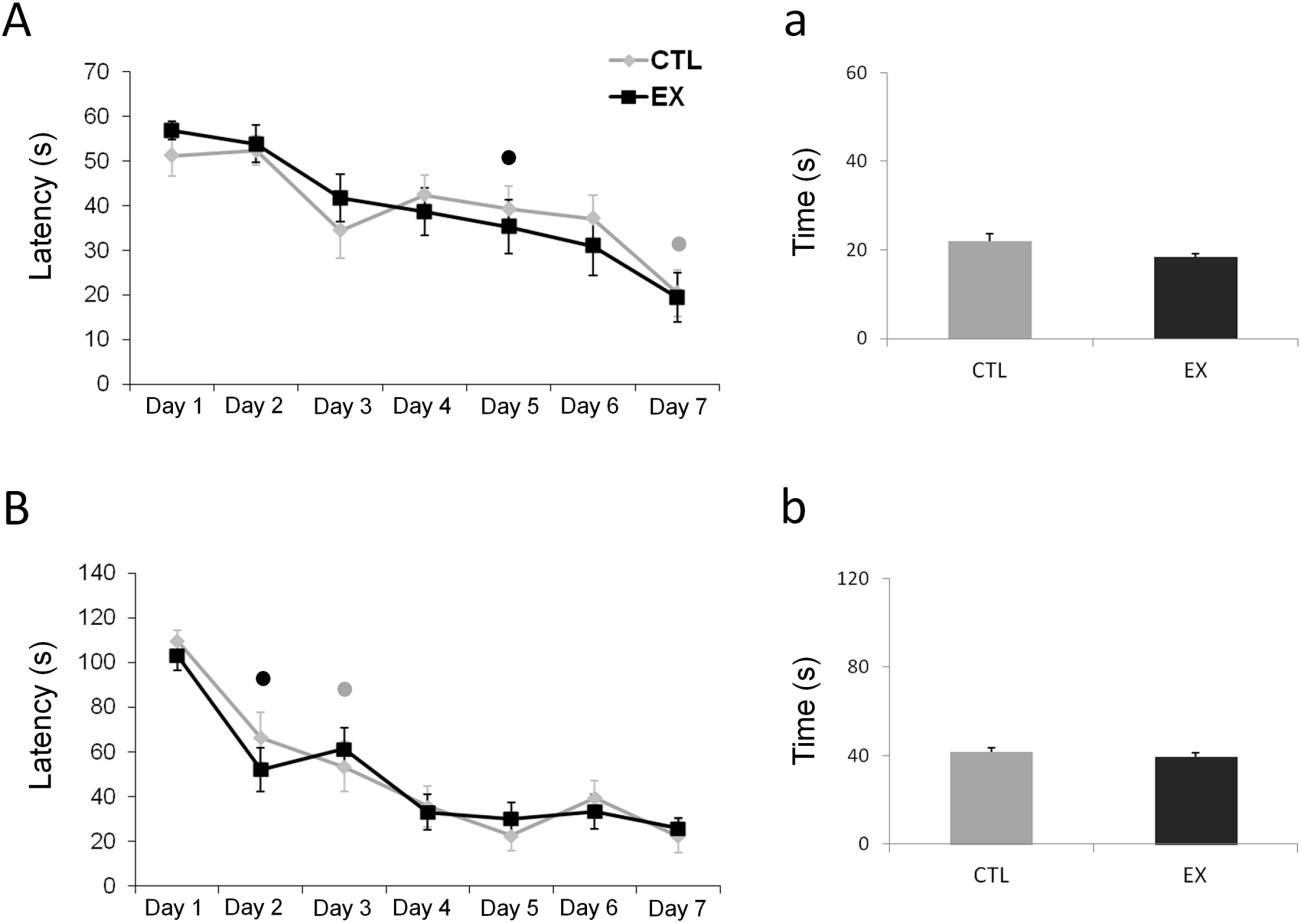
Spatial learning is faster in the offspring of exercised mothers. Latency of offspring from the exercise group (EX) and control group (CTL) to find the platform over 60 sec (n = 15 for EX group and n = 15 for CTL group) (A) and 120 sec (n = 15 for EX group and n = 15 for CTL group) (B) in the water maze test. No significant differences were detected between groups in either the 60-sec or 120-sec test (p > 0.05; one-way ANOVA with repeated measures). However, when analyzing the learning curve from each group separately, it was noted that offspring from the EX group exhibited better performance at finding the platform than those from the CTL group. In the 60-sec test (A), a statistical difference in comparison to the first test day emerged on the fifth day of testing for the EX group (•) and on the seventh day of testing for the CTL group (•). In the 120-sec test (B), a statistical difference in comparison to the first day emerged on the second day for the EX group (•) and on the third day for the CTL group (•). One day after the end of the last session in the water maze, the platform was removed (from quadrant 3) for a probe trial (a and b). Offspring were allowed to swim freely for 60 sec (a) or 120 sec (b), and time spent in quadrant 3 was used to assess offspring memory of the platform location. No significant difference in retention of this information was detected between the EX and CTL groups in 60 sec or 120 sec of investigation.

Spatial learning and memory were assessed in a water maze for seven consecutive days. One-way ANOVA with repeated measures showed that the latency in finding the platform over 60 sec or 120 sec decreased progressively with more test days (F_(6,168)_ = 11.931; p < 0.001 for the 60-sec trial; F_(6,168)_ = 23.63; p < 0.001 for the 120-sec trial) (Fig 5A and 5B). No significant differences were detected between groups for either 60 sec (F_(1,28)_ = 0.000; p = 0.983) or 120 sec (F_(1,28)_ = 0.143; p = 0.708). However, when analyzing the learning curve from each group separately, a statistical difference in comparison with the first test day emerged on the fifth day of testing for exercise group animals (p = 0.046) and on the seventh day of testing for control animals (p < 0.001) in the 60-sec test. In the 120-sec test, a statistical difference in comparison with the first day emerged on the second day for exercise group animals (p = 0.022) and on the third day for control group animals (p < 0.001). Altogether, these data indicate that animals in the exercise group were more rapid learners than those in the control group.

One day after the end of the last session in the water maze, the platform was removed for a probe trial. Animals were allowed to swim freely for 60 sec or 120 sec. Time spent in quadrant 3 was considered a measure of offspring memory of the platform location. No difference in retention of this information was detected between exercise and control groups in 60 sec (t_(28)_ = 1.992; p = 0.06) or 120 sec (t_(28)_ = 0.848; p = 0.4028) (Fig 5a and 5b).

### Aversive Memory

**Fig 6:**
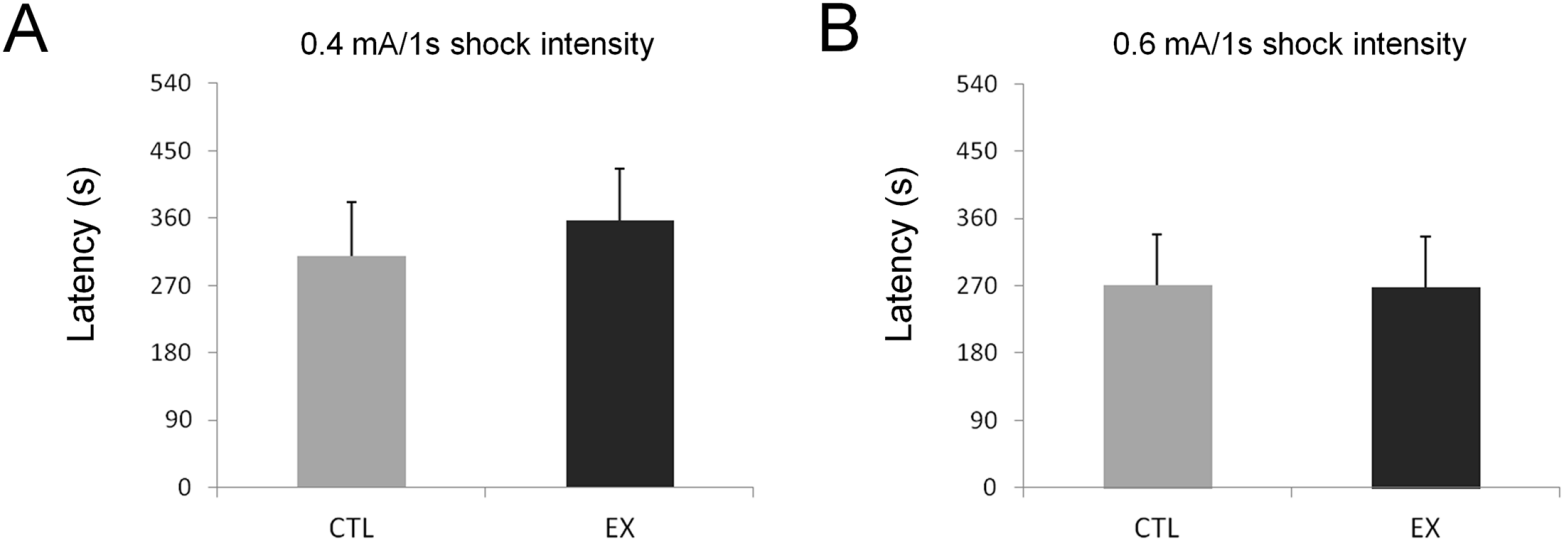
Aversive memory of offspring is not influenced by mothers’ exercise during pregnancy. Latency of offspring from the exercise group (EX) and control group (CTL) 24 h after they have been subjected to one of two footshock intensities: 0.4 mA for 1 sec (n = 14 for EX group and n = 13 for CTL group) (A) or 0.6 mA for 1 sec (n = 15 for EX group and n = 15 for CTL group) (B). No statistical difference in aversive memory was found between groups for either shock intensity (p > 0.05 by Student's *t* test).

Aversive memory was tested 24 h after the offspring was subjected to one of two footshock intensities (0.4 mA for 1 sec or 0.6 mA for 1 sec). Data analysis showed no statistical difference between the exercise and control groups for both the 0.4 mA (t_(25)_ = 0.485; p = 0.63) and 0.6 mA (t_(28)_ = 0.036; p = 0.97) shock intensities (Fig 6).

## Discussion

The present study assessed some molecular and cellular changes in the hippocampal formation and cerebral cortex of rats born from mothers submitted to treadmill exercise during pregnancy. Results showed that exercise during pregnancy induces long-term neuroplastic effects on BDNF levels and absolute cell numbers in the brain of offspring rats, namely in the hippocampal formation, a brain region important for memory, learning, and emotional functions. In addition, offspring from exercised mothers had better cognitive performance in nonassociative (habituation) and associative (spatial) mnemonic tasks in comparison with offspring from sedentary mothers.

Previous studies have described numerous beneficial effects of maternal exercise on cognitive function of offspring [4,5,6,18]. In our study, offspring of exercised mothers exhibited improvements in habituation behavior and spatial learning when compared to offspring of sedentary mothers (see Figs 4 and 5). However, no difference between studied groups (exercise and control) was found on exploratory activity during 5 or 10 min of open field locomotion (Fig 3), on spatial memory over 60 or 120 sec in a water maze (i.e., although exercise has learned more faster than control, no statistical difference between the groups was observed) (Fig 5), or on aversive memory with 0.4 mA or 0.6 mA footshocks of 1 sec duration in an inhibitory avoidance task (Fig 6). These last results diverge from previous studies, possibly due to the maternal exercise protocols used or to the age of the studied pups. For example, in the study conducted by Kim and co-workers [5], pregnant rats ran on a treadmill at an intensity of 5–8 m/min for 30 min/day over a period of 7 consecutive days from the 15^th^ to the 21^st^ gestational day (G15-G21), the last stage of fetal development. In our exercise protocol, pregnant rats ran on a treadmill at an intensity of 10–12 m/min for 30 min/day over 20 consecutive days (G1 to G20) spanning all of fetal development. Furthermore, we began to test the cognitive performance of offspring at P60, while other investigators began to test it at P28 [4,5], P36 [6], or P40 [3].

Nevertheless, it is important to point out that results similar to ours have been reported [19,20]. As mentioned above, we did not observe differences in exploratory open field activity between offspring in the control and exercise groups (Fig 3). Other authors, assessing exploratory activity of male pups during 5-min periods of open-field locomotion, also found no differences between offspring of exercised and sedentary mothers [19]. In a water maze test, we showed that animals in the maternal exercise group were faster learners than were control animals. However, in a probe test, no significant change in retention of spatial memory was found between them (Fig 5). Likewise, Akhavan and co-workers [20] reported that maternal exercise during pregnancy enhanced spatial learning of offspring in a water maze test (a 60-sec trial daily for 5 days) but not their retention of spatial memory.

In the present study, we observed higher hippocampal BDNF levels in the offspring of exercised mothers. This finding supports previous work showing that exercise during pregnancy is able to elevate BDNF mRNA expression in the hippocampal formation of offspring at P0 [3] and P29 [4,5]. Considering that BDNF is synthesized by both neurons and glial cells to regulate cellular processes of proliferation, development, and differentiation [21], it is probable that the higher hippocampal BDNF levels observed in our study might contribute, at least in part, to accelerated neurogenesis and thus to the increased absolute number of neuronal and non-neuronal cells observed in the hippocampal formation of animals in the exercise group. Additionally, increased offspring BDNF levels induced by maternal exercise could also be associated with the improvements in brain function (i.e., in habituation and spatial learning) found in our study. In favor of this idea, it has been observed that improvement of spatial learning in pups from exercised mothers was eliminated when the action of BDNF was inhibited in the hippocampal formation of pups [20].

Despite the findings described above, it is not known yet how exercise in the dam could impact BDNF in the offspring. A possible explanation for the increment of BDNF in the hippocampal formation of offspring could be attributed to exercise-induced epigenetic changes. Indeed, it has been noted that physical exercise is capable to change the gene expression that regulates histone acetylation and DNA methylation in the mice brain [23,24]. After one week of exercise, hippocampal DNA methylation status was found altered in BDNF gene promoter region [23]. In another study, global acetylation of histone 3 was increased and, surprisingly, this alteration was correlated with high levels of BDNF in the formation hippocampal of physically active mice [24].

As mentioned, maternal exercise during pregnancy significantly increased the absolute numbers of neuronal and non-neuronal cells in the hippocampal formation of the offspring. Besides BDNF, other factors might also contribute to this effect. Previous studies have shown that different models of maternal exercise induce profound effects on cell proliferation and survival [4,5,22,25]. It has been reported that pups born to rodents submitted to swimming, treadmill, or voluntary wheel running during pregnancy had more cell proliferation in the hippocampal region, as assessed by BrdU labeling, than did control pups [4,5,22]. Interestingly, this effect was also found when specific neurogenesis (BrdU/NeuN-double-labeled cells) and gliogenesis (BrdU/S100β-double-labeled cells) markers were used [22], suggesting that maternal exercise promotes the proliferation of both neuronal and non-neuronal cells in the brains of offspring.

In our study, no molecular (BDNF) or cellular (cell number) changes were detected in the cerebral cortex of offspring of the exercise group. These results are interesting and intriguing, because divergent findings have been described [18,19]. For instance, Uysal and collaborators [19] found higher levels of cortical BDNF in rat pups from exercised mothers with maternal deprivation but not in pups from exercised mothers without maternal deprivation. On the other hand, in a subsequent work [18], the same group described an increase in BDNF levels in pups from exercised mothers without maternal deprivation. Altogether, these findings indicate the need for further investigation of the influence of exercise during pregnancy on cortical plasticity in offspring.

In conclusion, our results indicate that maternal exercise during pregnancy enhanced offspring cognitive function (habituation behavior and spatial learning) and increased BDNF levels and cell numbers in the hippocampal formation but not in the cerebral cortex of adult rat offspring. Future studies should examine the epigenetic mechanisms underlying these changes, and investigate whether the beneficial effects of maternal exercise might be extrapolated to humans and in conditions that lead changes in the dynamics of the placenta, such as maternal epilepsy, fetal growth restriction, intrauterine inflammation and others.

## Supporting Information

S1 Table All original data. (XLSX)
